# Brazilian higher education system at the crossroads: the undergraduate student mental health crisis

**DOI:** 10.1016/j.lana.2026.101617

**Published:** 2026-09-01

**Authors:** Lauro Miranda Demenech, Beatriz Schmidt, Flavio Kapczinski, Luiz Eduardo Maia Nery, Marcia Maria Tavares Machado, Raquel Brandini de Boni, Sandra Brignol, Sonia Maria Lemos

**Affiliations:** a Center for Studies on Risk and Health, Federal University of Rio Grande (FURG), Rio Grande, Brazil; b Department of Psychology, Federal University of Health Sciences of Porto Alegre (UFCSPA), Porto Alegre, Brazil; c Department of Psychiatry and Legal Medicine, Federal University of Rio Grande do Sul (UFRGS), Porto Alegre, Brazil; d Institute of Biological Sciences, Federal University of Rio Grande (FURG), Rio Grande, Brazil; e Department of Community Health, Federal University of Ceará (UFC), Fortaleza, Brazil; f Institute of Scientific and Technological Communication and Information in Health, Oswaldo Cruz Foundation (Fiocruz), Rio de Janeiro, Brazil; g Department of Epidemiology and Biostatistics, Fluminense Federal University (UFF), Niterói, Brazil; h School of Health Sciences, Amazonas State University (UEA), Manaus, Brazil

**Keywords:** Mental health, Mental disorders, Higher education, Undergraduate students, Brazil

## Abstract

In a diverse and developing country, higher education is often viewed as a critical pathway to social mobility and national development. Although universities worldwide are facing a student mental health crisis, Brazil and other countries from the Global South present unique structural and cultural features that add layers of complexity to this growing concern. In this Personal View, we examine the landscape of undergraduate student mental health in Brazil by tracing its roots in the country's higher education history and its contemporary consequences. We focus on the opposing dynamics of sector expansion and democratization of access since the 2000s, followed by intensified underfunding from the mid-2010s. We discuss how academic migration, growing ethnic/racial and socioeconomic diversity, financial and human resource constraints, and generational shifts have created demands that exceed institutional capacities. We identify key research gaps and conclude with a call to strengthen research, address structural determinants, and promote student mental health.

## Resumo

Em um país diverso e em desenvolvimento, o ensino superior é frequentemente visto como um caminho central para a mobilidade social e o desenvolvimento nacional. Embora universidades em todo o mundo estejam lidando com uma crise de saúde mental entre estudantes, o Brasil e outros países do Sul Global apresentam características estruturais e culturais próprias que tornam esse cenário ainda mais complexo. Neste *Personal View*, analisa-se a saúde mental de estudantes de graduação no Brasil, resgatando suas raízes na trajetória histórica do ensino superior no país e suas implicações no contexto atual. São enfatizadas as dinâmicas contrastantes de expansão do sistema e democratização do acesso a partir dos anos 2000, seguidas por um período de crescente subfinanciamento desde meados da década de 2010. Discute-se como a migração acadêmica, a ampliação da diversidade étnico-racial e socioeconômica, as limitações de recursos financeiros e humanos e as mudanças geracionais têm gerado demandas que superam a capacidade de resposta das instituições. Por fim, são destacadas lacunas relevantes na produção científica e apresenta-se uma chamada à ação para fortalecer a pesquisa, enfrentar os determinantes estruturais e promover a saúde mental dos estudantes.

## Introduction

Every student at the university embodies multiple layers of investment, including their own dedication, the support and resources provided by their families, and the financial and structural commitments made by both public and private sectors. These investments are made with the expectation that higher education will generate returns not only in terms of individual socioeconomic mobility, but also in collective gains such as technological innovation, scientific progress, and improved public health. At the same time, university life coincides with a critical developmental stage in which young adults are more vulnerable to the onset of mental disorders.^1^ In this setting, universities play a dual role: they may exacerbate stressors that trigger or worsen psychological distress, but they can also function as a unique window of opportunity to promote resilience and positively influence life trajectories.

Higher education systems vary significantly across countries, each with distinct structural and cultural characteristics. Nevertheless, much of the global discourse on university student mental health relies on research and experiences rooted in high-income countries. While there are indeed shared features worldwide that contribute to a global student mental health crisis, such as developmental vulnerabilities, generational forces, and academic pressures,^2^ the Brazilian context, along with that of other Latin American countries, presents a specifically complex landscape. They are shaped by structural inequalities, historical exclusion, and the still incomplete democratization of higher education, elements that are crucial to understanding the current scenario. Therefore, this article provides a comprehensive analysis of the history, organization, and current state of Brazilian higher education, examining how political, economic, demographic, and cultural changes may affect students' mental health. Published studies, along with original and official data, were analyzed to offer an overview, identify research gaps, and propose future directions for both investigation and mental health care strategies.

## Search strategy and selection criteria

We searched PubMed for articles on mental health outcomes among Brazilian undergraduate students from database inception to April, 2025. The search strategy included the following MeSH terms in various combinations: “Mental Health”, “Mental Disorders”, “Depression”, “Anxiety”, “Stress, Psychological”, and “Brazil”. We also used the text words “University Students”, “College Students”, and “Undergraduate Students” to define the target population. No language restrictions were applied. References were selected based on their relevance and originality to inform this Personal View.

## Higher education in Brazil

Brazil is one of the largest and most populous countries in the world, ranking fifth in territorial extension and seventh in population, with more than 200 million inhabitants and a diverse cultural, ethnic, demographic, and socioeconomic background.^3^ This vast and diverse nation, however, experienced a unique trajectory in the development of its higher education institutions (HEIs).

The structure and mission of Brazilian higher education have evolved through distinct political and social regimes. Panel 1 shows a brief history of the development of higher education in Brazil. Unlike Spanish colonies, where universities were established as early as the 16th century, the Portuguese colonial model in Brazil delayed the emergence of higher education until the 19th century. Therefore, its roots were oriented toward the specialization and training of the country's economic and political elite, reinforcing the *status quo*. Over the 20th century, cycles of reform, repression during military dictatorship, and privatization shaped a fragmented and unequal landscape. Following Brazil's political redemocratization and the 1988 Constitution, tuition-free public universities were guaranteed, although a dual structure remained: public HEIs, offering tuition-free education at the federal, state, and municipal levels, funded through national, state, and municipal tax revenues; and private HEIs, which charge tuition fees and include both for-profit and non-profit institutions (such as religious, community, and philanthropic organizations), primarily funded through tuition payments.^4^

Panel 1 **The historical trajectory of Brazilian higher education and its relevance to student mental health.**

**Colonial legacy and elite foundations**

Portuguese colonial model did not prioritize higher education. Therefore, the first HEIs in Brazil have emerged only in the 19th century, following the arrival of the Portuguese Royal Family. By the end of the Empire of Brazil (1889), the country had only six HEIs, focused on training elites for legal, medical, and engineering professions to serve the Portuguese court.^4^^,^^5^

**Fragmentation and early reforms**

In the early 20th century, Brazilian higher education was composed of isolated, vocational schools with limited scientific orientation. However, it was during this period that the first universities, as we know them today, were established, such as the University of Amazonas (1909), the Federal University of Paraná (1912), and the University of Brazil (now the Federal University of Rio de Janeiro, 1920). The Francisco Campos Reform in 1931 (named after the country's first Minister of Education) sought to regulate university operations, although public education was not yet tuition-free. The subsequent decades were marked by ideological disputes over the role of the state versus the private sector in education. One of the outcomes of this period was the establishment of Catholic universities, such as the Pontifical Catholic University of Rio de Janeiro (PUC-RJ) in 1940.^6^ This debate was formalized in 1961 with the first National Education Guidelines and Framework Law, which favored private sector participation in higher education while maintaining the presence of public institutions.^7^

**Military rule and institutional modernization**

During the military dictatorship (1964–1985), student movements were suppressed, and public universities came under surveillance. Despite political repression, the 1968 University Reform modernized the system by integrating teaching, research, and community engagement. However, these comprehensive institutions became increasingly expensive, and growing demand led to the expansion of private HEIs. The increasing authorization of new institutions, beginning in the 1970s, was granted as long as they met minimal financial and human resource requirements, raising concerns about the quality of education.^4^, ^5^, ^6^

**Democratization and structural duality**

With the return to democracy, the 1988 Federal Constitution was promulgated, a document that remains in force. One of the major changes in Brazilian higher education was the legal guarantee that public education would be free of charge (Article 206) while also allowing for private sector participation (Article 208).^8^ The 1996 National Education Guidelines and Framework Law consolidated a dual system: public, tuition-free HEIs funded by tax revenues, and private HEIs, both non-profit and for-profit, sustained primarily by student fees.^9^

**Access expansion in the 21st century**

The federal government introduced the National High School Exam (ENEM) in 1998, originally as an assessment of learning outcomes. By 2009, ENEM evolved into the central admission criterion for federal and many state HEIs through the Unified Selection System (SiSU). This centralized platform enabled students to compete for university places nationwide using a single score, reducing logistical and financial barriers. While this reform improved geographic and socioeconomic access, it also introduced new challenges as it fostered the inclusion of historically marginalized groups and intensified the academic migration of young individuals within a country as large as Brazil. These challenges include disparities in institutional infrastructure and student support services across regions, increased dropout rates, and growing demands for mental health and psychosocial support to address adaptation difficulties, financial stress, and social isolation.^10^, ^11^, ^12^ The role of ENEM in shaping these changes is discussed in greater detail in the main text.

**Contemporary consequences**

This historical trajectory produced a structurally unequal and fragmented higher education system. The rapid expansion of access was not matched by institutional preparedness or student support. Today, many students, particularly those from marginalized backgrounds, face academic pressures within institutions that are still shaped by exclusionary and colonial legacies, including elitist academic cultures, as well as persistent forms of social and racial discrimination. These structural conditions form an essential backdrop for understanding the mental health vulnerabilities observed among Brazilian undergraduates.

Traditionally, admission to public and private HEIs occurred through institution-specific entrance exams, a process criticized for perpetuating social inequalities. Students from wealthier backgrounds, typically educated in private schools, were disproportionately represented in the most prestigious universities.^10^^,^^11^ However, at the turn of the 21st century, Brazil implemented a series of reforms, legal frameworks, and programs aimed at expanding and sustaining access to higher education (Table 1).

**Table 1:** Government programs and initiatives for the expansion and democratization of access and permanence in Brazilian higher education. Brazil, 2025.

Program	Legal framework	Objective
1988 Public spending on education	Article 212 of the Federal Constitution.^8^	Established a minimum of 18% of federal government net tax revenue and 25% of states and municipalities net tax revenue for public spending on education
1998 ENEM–National High School Examination	Ministry of Education Ordinance No. 438, of 1998,^13^ reformulated by the Ordinance No. 109, of 2009.^14^	Established the National High School Examination (ENEM) as an assessment of secondary education. After 2009, it was reformulated and widely adopted as a unified mechanism for higher education admission.
2001 FIES–Student Financing Fund	Law No. 10,260 of 2001, last updated in Law No. 14,375 of 2022.^15^^,^^16^	Established the Student Financing Fund intended to grant financing to students of higher education programs in Private HEIs.
2005 ProUni–University for All Program	Law No. 11,096 of 2005, last updated in Law No. 14,350 of 2022.^17^^,^^18^	Established the granting of full or partial scholarships in Private HEIs for those who do not hold a higher education diploma, and come from low-income families.
2007 REUNI–Support Program for Restructuring and Expansion Plans of Federal Universities	Presidential Decree No. 6096 of 2007.^19^	Established the increase of resources allocated to Federal HEIs for the creation of new universities, infrastructure works, purchase of goods and services, expenses, and costs.
2010 PNAES–National Student Assistance Program	Presidential Decree No. 7234 of 2010.^20^	Established the expansion of the conditions for university students permanence in the areas of student housing, food, transportation, comprehensive health care, digital inclusion, culture, and pedagogical support.
2012 Quota Law	Law No. 12,711 of 2012.^21^	Established a 50% reserve of places in Federal HEIs for candidates who completed high school in the public education system. Within this 50% for public schools, there is a reserve of places according to characteristics that historically stratify Brazilian society concerning opportunities, such as race/ethnicity, low family income, and disabilities.

**Fig. 1:**
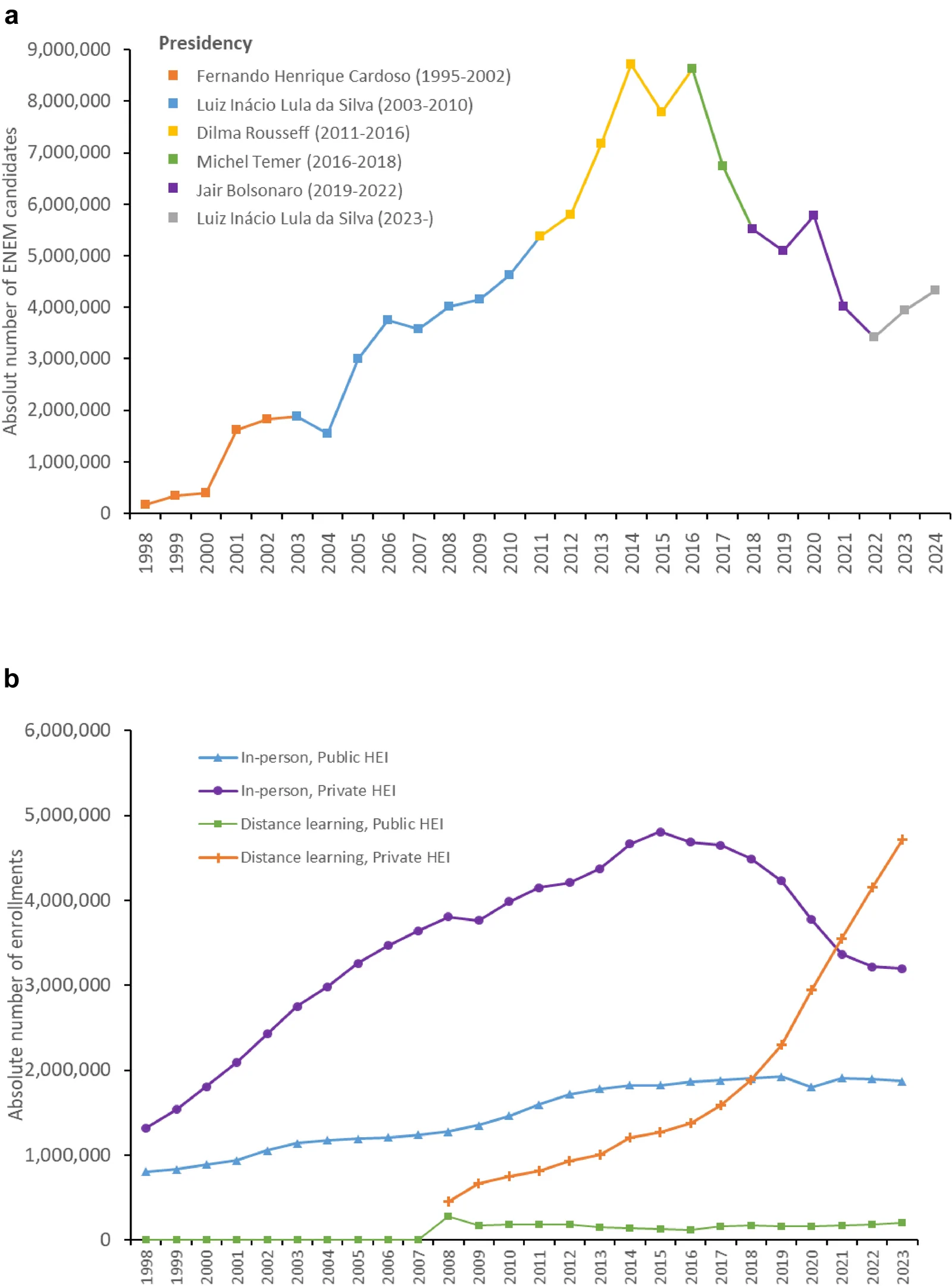
**a) Trajectory of applicants for the National High School Exam (ENEM) (1998–2024). b) In-person and distance learning undergraduate students enrolled in Brazil, stratified by type of HEI (Public or Private) (1998–2023).** Data obtained from the records of the Brazilian National Institute of Studies and Educational Research (*Instituto Nacional de Estudos e Pesquisas Educacionais Anísio Teixeira*–INEP). Brazil, 2025.^12^

The National High School Exam (ENEM) was created in 1998 to assess Brazilian students' performance at the end of basic education. Since 2009, the exam score has also been used as a unified mechanism for HEIs admission for undergraduate programs across the entire country, especially for public institutions, but also for some private HEIs. As shown in Fig. 1a, there was a significant increase in ENEM registrations, rising from 4,148,721 in 2009 to 8,722,290 in 2014.^12^

This surge in exam participation reflected a broader social climate of confidence and engagement with higher education. It was mainly driven by the prospect of social mobility created through political and financial incentives for higher education, particularly during the presidencies of Luiz Inácio Lula da Silva (2003–2010) and Dilma Rousseff (2011–2016). In addition to changes in the HEI admission process, several programs and policy initiatives have been implemented to support the expansion and democratization of access and retention in higher education, including the Student Financing Fund (FIES), the University for All Program (ProUni), the expansion of federal universities through REUNI, the National Student Assistance Program (PNAES), and the Quota Law, which introduced race- and income-based affirmative action in federal HEIs.

Nevertheless, the notion of social mobility through higher education is relatively recent, and the persistent historical, cultural, and social barriers embedded in the 200-year development of this system continue to underlie many of the challenges still faced by Brazilian universities today, and must be considered in any discussion of mental health in this population.

## Changing student demographics and emerging challenges

As a consequence of the democratization strategies, there was a change in undergraduate students' profile. The ENEM/SiSU model provided a higher probability of admission in HEIs far from the original students' place of residency, increasing academic migration within Brazil. In contrast to many high-income countries, where leaving one's place of residence to attend university is often expected, undergraduate education in Brazil has traditionally been more locally oriented, with students remaining closer to family and established social networks. Academic migration at the undergraduate level represents a relatively recent phenomenon in Brazil. Students are 20·4% more likely to migrate to another state since this new selection model was adopted.^22^ This rise brings challenges beyond geographic relocation, as it also involves social, cultural, and climatic adjustments, factors that are intensified in a country as large as Brazil. The increased mobility among younger students can disrupt social support networks during a pivotal developmental period for the onset of mental disorders,^1^ contributing to experiences of isolation, financial strain, and adaptation difficulties, which may in turn increase the risk of dropout and adversely affect mental health. Although research on the health impact of academic migration within Brazil is still limited, few studies indicate that students who migrate longer distances are more likely to drop out^22^ and to use illicit substances.^23^

There was also an increase in socioeconomic diversity in higher education, becoming a closer reflection of Brazilian society. Between 2003 and 2010, the proportion of Black students increased from 5·9% to 8·7%, while the proportion of mixed-race (Pardo) students grew from 28·3% to 32·1%. Between 2002 and 2015, the proportion of young people aged 18–24 from the poorest quintile of the population attending higher education rose from 0·3% to 4·7%.^24^ A report from the National Association of Directors of Federal Higher Education Institutions (Andifes) on the socioeconomic profile of students indicates similar trends. In 2018, the majority of students in federal HEIs self-identified as Black or mixed-race (51·2%), with 70·2% of them having a per capita family income equal to or below 1·5 minimum wages.^25^ According to the latest Brazilian census, 45·3% of the population identified as mixed-race and 10·2% as Black.^3^ Furthermore, 60·1% of the population lives on up to one minimum wage.^26^ In addition to becoming more socioeconomically diverse and more representative of Brazil's demographic distribution, there has also been an increase in the participation of students from the North, Northeast, and Central-West regions, historically poorer regions compared to the South and Southeast, resulting from the establishment of new institutions in these regions through the REUNI program after 2007.^27^ However, these changes have not fully mitigated patterns of academic migration, as institutional prestige and uneven distribution of resources continue to shape student mobility across the country.

This demographic transformation represents a major achievement for Brazil, as it expanded higher education access to historically excluded populations,^10^ targeting the reduction of social inequalities in the long run. However, new challenges arise. Many universities were not fully prepared to accommodate a more socioeconomically diverse and geographically mobile student population, particularly in terms of infrastructure, student support services, and inclusive academic environments. First-generation and low-income students often face financial instability, academic difficulties linked to prior educational inequalities, and limited social support, increasing the risk of dropout.^27^^,^^28^ Additionally, growing academic migration intensifies social isolation, cultural dislocation, and adaptation stress.^10^ As a result, demand for mental health and psychosocial support has increased within universities, which frequently lack the resources and institutional capacity to respond adequately.^29^ This mismatch between expanded access and institutional preparedness represents a key emerging challenge for the sustainability and equity of higher education in Brazil.

## Change in budget policy and the erosion of public institutions

It is essential to analyze these trends in light of recent changes in federal budget policies for education, particularly higher education. As a result of the democratization strategies, the number of students enrolled in in-person undergraduate programs at public HEIs, those with the highest national and international relevance, doubled between 1998 and 2011, maintaining a steady growth rate until 2015, after which it stabilized (Fig. 1b). Enrollment in in-person undergraduate programs at private HEIs experienced even steeper growth, quadrupling between 1998 and 2015. Contrary to the democratization movement, austerity policies targeting public funding of the HEIs were implemented during Dilma Rousseff's administration (2015) and institutionalized under Michel Temer's government (2016–2018) with Constitutional Amendment 95/2016. This amendment imposed a ceiling on primary government spending, freezing it at 2017 levels for twenty years, resulting in a sustained decline in real investment in public universities.^27^^,^^30^

The process of institutional erosion was further intensified during the administration of Jair Bolsonaro (2019–2022), a period marked by substantial reductions in federal funding for science, technology, and public universities,^31^ which limited the capacity of institutions to sustain research and maintain basic operations and reinforced an ongoing trajectory of structural weakening.^31^^,^^32^ Beyond financial constraints, this period was also characterized by growing institutional uncertainty and reduced policy coordination, undermining the stability required for long-term academic planning and development.^32^ These challenges were compounded by the COVID-19 pandemic, during which Brazil experienced one of the highest mortality burdens globally,^33^ in a context marked by delayed and fragmented federal responses.

For roughly six years, the higher education system faced a progressive erosion of its financial structure, as expenditures on education fell not only in relative terms but also as a share of GDP, despite the historical pattern of investments above the constitutional minimum of 18%.^34^ Although the new fiscal framework established by Constitutional Amendment 126/2022 and Complementary Law 200/2023 replaced the previous spending cap, it maintains tight constraints on public expenditure growth, offering limited prospects for a substantial recovery of higher education funding.^35^

Underfunding weakens the structure of public HEIs, limiting their capacity for maintenance, creating barriers to teaching and research, hindering scientific and technological advancement, and discouraging public interest in higher education. Additionally, it favors the privatization process,^30^ as can be observed in the sharp rise in private distance learning enrollment in Fig. 1b. In Brazil, distance learning degrees are predominantly offered by private institutions and are often characterized by lower tuition costs and greater flexibility, increasing their appeal among students from lower-income backgrounds. This flexibility allows individuals to combine work and study, an option that is often not feasible in traditional full-time programs. However, the rapid expansion of low-cost private programs raised concerns regarding their quality, especially in relation to academic demands and labor market outcomes.^36^^,^^37^

The scenario envisioned in the early 2000s was that of a nation committed to social mobility through higher education, generating positive expectations for individuals, and their families, and the development of a strong state in science and technology.^5^ In contrast, today's outlook is far more pessimistic, characterized by chronic underfunding, which hampers the training of future professionals and discourages market growth and opportunities for these students. This abrupt shift in expectations over a short period may be significantly contributing to the current landscape of psychological distress observed in the Brazilian university context. Moreover, considering the historically elitist nature of Brazilian higher education, it is also plausible that university faculty and administration are not adequately prepared to understand and educate the new student profile, further exacerbating the issue.

## The undergraduate student mental health crisis in Brazil: what do we know?

The increasing concern surrounding Brazilian undergraduates' mental health must be contextualized within the global rise in mental health problems. It is estimated that more than one billion individuals worldwide live with mental disorders, most of whom reside in low- and middle-income countries. The global age-standardized point prevalence reached 13·6% in 2021, an increase of 0·9% since 2011, with young adults aged 20–29 years showing the largest rise (1·8%).^38^ Among university students, the situation is even more concerning. The *World Mental Health International College Student Initiative*, which surveyed 72,288 first-year undergraduates from 18 countries across five continents, found that nearly two-thirds (65·2%) had experienced an internalizing or externalizing mental disorder at some point in their lives, while over half (57·4%) screened positive in the past 12 months.^1^ The burden is both high and rising: data from the *Healthy Minds Study*, an annual survey of U.S. university students, indicate substantial increases between 2013 and 2025 in depression (22·3%–37·5%), anxiety (17·2%–31·9%), and suicidal ideation (8·3%–11·3%).^39^^,^^40^ These alarming results highlight a mental health crisis in undergraduate education.^2^

Despite university students being a widely studied population, Brazil lacks robust and standardized nationwide initiatives aimed at investigating university students' mental health. Existing literature on the subject is characterized by small-scale, regionally focused studies with high heterogeneity in measurement methods, making comparisons and a broader understanding of the scenario difficult.^41^^,^^42^ Nevertheless, a meta-analysis of studies published up to January 2020, including students from all academic fields, reported pooled prevalence rates of 37·8% for anxiety, 28·5% for depression, and 9·1% for suicidal behaviors.^41^ Higher depression rates were found in studies conducted in public universities and in samples composed exclusively of medical students, while anxiety was more frequent in studies using probability sampling strategies.^41^

The COVID-19 pandemic was an unprecedented global health crisis that profoundly affected universities and students.^43^^,^^44^ Several Brazilian studies evaluated its psychological effects on undergraduate students by screening mental health problems and their associated factors. At a public university in the state of Goiás, 33·4% of students exhibited depressive symptoms, with associations found between these symptoms and moderate to high-risk patterns of alcohol, tobacco, and illicit drug use.^45^ In the state of Rio Grande do Sul, a study involving the academic community of a public university (including students, staff, and faculty) found that 39·2% of participants screened positive for depression, and 52·5% for anxiety. Among all subgroups, undergraduate students exhibited the highest prevalence rates (49·1% for depression and 60·5% for anxiety). The study identified key risk factors, including social isolation, lack of psychological support, and a history of mental disorders.^46^ Additionally, Demenech et al.^47^ carried out a two-stage study between 2019 and 2021. In the first stage, conducted at a public university in southern Brazil, an increase was observed in the prevalence of depression (from 38·3% to 54·7%) and suicide risk (from 11·3% to 17·0%) between 2019 and 2020. In the second stage, conducted during the pandemic across universities from all five regions of Brazil, the prevalence rates of positive screening for anxiety, depression, and suicide risk were 36·0%, 55·2%, and 19·3%, respectively.

**Fig. 2:**
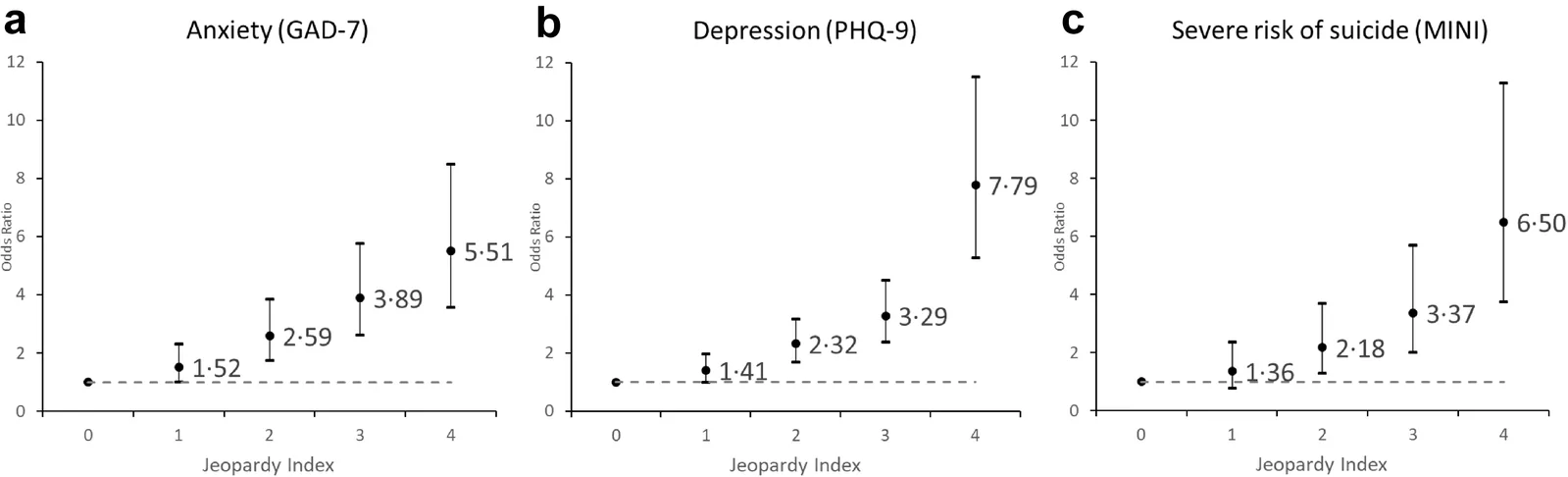
**Odds ratios of positive screens according to Jeopardy Index levels: (a) anxiety (GAD-7), (b) depression (PHQ-9), and (c) severe risk of suicide (MINI).** Results from the Health and Wellness of Undergraduate Students (SABES-Grad) Study, Brazil, 2021.^52^

The factors associated with higher prevalence rates of mental disorders among students are similar to those observed in the general population, including belonging to social minorities, experiencing socioeconomic inequalities, having a previous mental health diagnosis, and having a family history of mental disorders.^46^^,^^48^, ^49^, ^50^, ^51^ Brazilian HEIs have mindfully increased access to a more diverse student population, mirroring the country's demographic. Fig. 2 depicts the cumulative impact of intersectional vulnerabilities on the likelihood of anxiety, depression, and severe suicide risk, based on data from the SABES-Grad Study, a multicentric survey of 4994 undergraduates across Brazil conducted in 2021.^52^ The Jeopardy Index was calculated by summing four jeopardy markers: sex assigned at birth (male = 0, female = 1), race/ethnicity (white = 0, mixed-race/Black/other = 1), sexual orientation (heterosexual = 0, homo-/bi-/pansexual = 1), and per capita family income (highest quintile = 0, other quintiles = 1). Anxiety, depression, and severe suicide risk were assessed with the Generalized Anxiety Disorder-7, Patient Health Questionnaire-9, and Mini International Neuropsychiatric Interview, respectively (methods, variables, and cutoff scores are fully described elsewhere^52^). Students with no vulnerabilities (white, heterosexual, male, highest income) served as the reference group. A clear dose–response relationship was observed: the odds of psychological problems increased with higher Jeopardy Index scores. The strongest associations were found among those with all four vulnerabilities (mixed-race/Black/other, homo-/bi-/pansexual, female, lowest income), with odds ratios of 5·51 (95% CI 3·57–8·49) for anxiety (Fig. 2a), 7·79 (95% CI 5·27–11·50) for depression (Fig. 2b), and 6·50 (95% CI 3·74–11·28) for severe suicide risk (Fig. 2c). Similar results were also found by Lima et al.,^53^ reflecting the cumulative effects of social inequalities on mental health. These findings align with intersectionality theory, which argues that multiple social identities interact within broader systems of inequality to shape health outcomes.^54^^,^^55^ Accordingly, the observed gradients likely reflect the compounded effects of overlapping forms of social disadvantage rather than isolated risk factors.

Additionally, the academic environment can impose high demands on students, with elevated stress levels being associated with an increased incidence of depression.^50^ Dissatisfaction with one's academic program has been frequently associated with various negative mental health outcomes.^42^^,^^48^^,^^49^ Furthermore, psychological distress has been linked to studying in a capital city, difficulties adapting to the university's location, and challenges in making friends.^41^

## Unanswered questions in Brazil: research gaps and paths forward

Brazil faces a significant challenge in measuring and quantifying mental health disorders among university students. The country lacks a nationwide probability sample study that evaluates psychiatric diagnoses in this population, rather than screening for mental health symptoms. In addition to the use of screening tools, the reliance on convenience samples in many published studies may lead to over- or underestimations due to selection and classification biases. There is also very limited information on the mental health status of the growing population of distance-learning students enrolled in private institutions. These gaps in research makes it difficult to determine the true prevalence of mental disorders among this population, hampering the allocation of resources and decision-making.

The second gap concerns the limited understanding of how variables related to the higher education system influence and exacerbate mental health problems among undergraduate students. The candidate selection model introduced in 2009 via ENEM/SiSU may influence mental health in several ways. The application period now lasts one week, with daily updates on the cutoff scores for all spots available in the unified system. Candidates can change their choice until the last day, making admission more accessible. However, the possibility of selecting an undergraduate program based on what was achievable rather than true interests or aptitudes may lead to greater difficulties in adjustment. Both course dissatisfaction and poor academic performance have been associated with negative mental health outcomes.^56^^,^^57^ The increase in the proportion of within-country migration may also play a role, especially in a large country such as Brazil. Migration during adolescence and young adulthood has been linked to an increased risk of psychosis, especially among racial minoritised populations.^58^ There is a need for a better understanding of these migration patterns and their effects on undergraduates' mental health.

The third gap concerns whether the high levels of psychological distress reported by various studies indicate that the issue is worsening, as empirically perceived by those working within this context. There is a lack of consistent time-series investigation on this topic in Brazil (such as the Healthy Minds Study in the U.S.). It is also not possible to firmly state that mental health among undergraduate students is worse than the general population. Research conducted in England showed no difference in rates of common mental disorders, suicide attempts, and non-suicidal self-harm between university students and their same-age non-student counterparts.^59^ Considering the increased diversity in Brazilian higher education, it is possible that the prevalence of mental disorders among undergraduates simply reflects the general population's mental health trends, with universities being unprepared to meet these demands. It would also be important to understand the generational aspects contributing to this scenario. There is evidence that Gen Z individuals, those in the age group more prevalent in universities nowadays, present the worst rates of mental health indices among the generational groups.^60^ Perhaps there is a mismatch between what this newly included student population encounters in terms of university demands, and their academic preparedness from prior education (also considering the COVID-19 pandemic impacts in a sensitive developmental stage), ultimately leading to psychological distress.

The fourth gap refers to the absence of longitudinal studies assessing how much of the burden of psychological problems originates during higher education. It is estimated that the median age of onset of mental disorders among undergraduates worldwide is 16 years, which suggests that much of the burden observed within universities is related to factors predating higher education entry.^1^ We identified one study that followed 1034 Brazilian undergraduates for three years (2015–2017), reporting a cumulative incidence of 28·3% and an incidence rate of 21·2 per 100 person-years of depressive symptoms.^50^ However, the most important predictors of depression onset among first-year students included prior suicide plans or attempts, a history of childhood or adolescent trauma, stressful experiences in the past 12 months, parental psychopathology, and the presence of other recent mental disorders.^61^

The last and equally important gap regards the opposing political and economic movements of the early 2000s (expansion of higher education and social mobility through education) and the mid-2010s (funding cuts that strengthened the private sector and weakened public universities). These contextual-level and broader determinants may have affected students' mental health.^62^ Future investigations should include a multilevel perspective to address whether the universities' difficulties in meeting students' psychological needs can be attributed to a lack of intersectoral resources not only directly related to education itself, but also social assistance, transportation, leisure, and food services.

## Call to action

Although there is still a long way to go before achieving full democratization of access to higher education in Brazil, some progress has been made in recent decades, as previously discussed. However, this progress also brings new challenges, such as the lack of preparedness from universities' side to welcome and support the increasing participation of students from lower socioeconomic backgrounds in higher education, as well as the intensification of academic migration within the country. These factors may increase the proportion of university students exposed to financial and food insecurity, inadequate housing conditions, transportation and leisure difficulties, discrimination, and barriers to healthcare access. This combination of challenges may create a vulnerability framework that affects students' mental health.^62^ This new scenario places university administrations in a strategic position to address mental health issues, as it has become a crucial matter for university management.^63^ Given this context, we propose three lines of action: *strengthening the research agenda*, *addressing the structural determinants of student mental health*, and *promoting mental health well-being, prevention, and care strategies*.

### Strengthening the research agenda

It is essential for research on Brazilian university students' mental health to advance through robust studies capable of answering the challenging questions outlined in the previous section of this article. A strong research agenda depends on financial investment, but historically very few resources have been allocated to such investigations. Recent developments suggest a possible shift in this trend. In 2023, the National Council for Scientific and Technological Development (CNPq) Call 24/2023 allocated U$ 1·5 million to establish a national mental health research network aimed at estimating the prevalence of selected mental health disorders within the university community (faculty, students and staff) and comparing it with the general population.^64^ This is an essential starting point, but sustained investments in monitoring mental health disorders across the universities are going to be necessary.

Interinstitutional collaboration can be a key strategy in achieving these goals. Brazil's higher education system is vast, complex, and regionally diverse, making it crucial to incorporate multiple perspectives and ensure research extends beyond the traditional South–Southeast axis.^41^^,^^42^^,^^65^ Additionally, it is recommended that research projects actively involve various stakeholders, including students, faculty, administrative staff, and university management.

### Addressing the structural determinants of student mental health

Addressing the mental health challenges faced by university students in Brazil requires tackling the structural determinants that shape these outcomes. The remarkable expansion of enrollment and student diversity has not been accompanied by proportional investments in institutional capacity or mental health infrastructure, leaving support services limited, fragmented, and overburdened.^29^ Like other developing countries, Brazil faces chronic underinvestment in mental health and a shortage of specialized professionals.^66^ These challenges are further exacerbated within underfunded university settings, resulting in significant inequalities in students' access to mental health services both on- and off-campus.^67^

Revisiting Brazil's fiscal framework is crucial to ensure that public higher education can recover from years of austerity and regain sustainable funding. Policies that support student permanence, including access to adequate housing, food security, transportation, and financial assistance, are essential to mitigate the social and economic stressors disproportionately affecting students from lower-income backgrounds. In parallel, universities must actively promote inclusive and supportive environments by addressing discrimination and fostering a sense of belonging among increasingly diverse student populations.

Strengthening student support structures is also essential, including expanding their capacity to provide care, developing preventive and health promotion actions, and reinforcing their integration with Brazil's Unified Health System (SUS) and Unified Social Assistance System (SUAS). These efforts should be articulated within a coordinated national strategy, which could be achieved by establishing a National University Mental Health Policy. Defining the roles of universities, health systems, and social services, as well as engaging key stakeholders at institutional and municipal levels, will be essential to ensure effective implementation and sustainable resource allocation.^68^

### Promoting mental health well-being, prevention, and care strategies

University entrance period frequently coincides with a developmental stage more sensitive to the development of psychological problems, therefore being a window of opportunity for prevention and early treatment of mental disorders.^69^^,^^70^ In a review of evidence regarding the effectiveness of interventions delivered within the university context, it has been shown that cognitive-behavioral therapy, mindfulness-based interventions and recreation programs (e.g., physical activities, yoga, and arts) were effective to reduce common mental disorders symptoms. However, cognitive-behavioral therapies showed the best enduring benefits over time.^70^

Considering the challenging scenario, it is also necessary to develop and implement innovative strategies to deliver such mental health care. One potential approach is the task-sharing model, which consists of psychosocial interventions delivered by non-specialist community providers and was designed for low-resource settings.^71^ These strategies typically involve training community members in brief, simple, and effective interventions to address common mental disorders.^72^ Through training and specialized supervision, the number of individuals capable of providing psychological support for high-prevalence conditions increases. Brazilian universities may offer a fertile ground for implementing this stepped model of care, tailoring limited resources according to case severity. This system allows for the assessment of case complexity, ensuring that specialists, such as psychiatrists and psychologists, focus on severe cases, while those with less complex needs may receive low-intensity interventions. Additionally, given the increasing diversity of the university population, these practices can provide culturally and socially sensitive care, thereby enhancing both coverage and access to mental health services.^73^ Mental health interventions delivered via technology also seem to be feasible and effective,^70^^,^^74^^,^^75^ increasing institutions' capacity to screen cases more efficiently and to deliver psychological interventions.

## Concluding remarks

We must reaffirm the vision for higher education that Brazilian society desires. If we choose to support a strong and democratic university system, as advocated in this article, we cannot afford to stop halfway. A higher education system cannot be sustained by good intentions alone. We are facing a mental health crisis in undergraduate education that can only be addressed with courage, responsibility and resources. If we aspire to build a strong and sovereign country with development, technology, and well-being, we must reinvest in improving university infrastructure, equipping faculty to meet contemporary demands, and enhancing student training. Ultimately, it would strengthen our capacity to prevent, identify, and provide care for students in need, thereby reducing the mental health burden in this population. Beyond Brazil, amplifying Latin American and Global South perspectives in this debate is essential. Recognizing education and mental health as fundamental human rights requires context-specific approaches that account for structural inequalities, cultural factors, and educational systems. Only then will global responses be equitable and truly inclusive.

## Contributors

LMD and BS–Conceptualization, data curation, investigation, project administration, visualization, writing-original draft, and writing–review & editing. FK, LEMN, MMTM, RBDB, SB, and SML–Conceptualization, data curation, investigation, project administration, visualization, and writing–review & editing.

## Declaration of interests

We declare no competing interest.

## References

[1] Mason A., Rapsey C., Sampson N. (2025). Prevalence, age-of-onset, and course of mental disorders among 72,288 first-year university students from 18 countries in the World Mental Health International College Student (WMH-ICS) initiative. J Psychiatr Res.

[2] Demenech L.M., Duffy A. (2026). Student mental health is in crisis - here's how to help. Nature.

[3] Instituto Brasileiro de Geografia e Estatística (IBGE) (2023). 2022 Population census: overview. https://censo2022.ibge.gov.br/panorama/.

[4] Neves C.E.B., Martins C.B., Dwyer T., Zen E.L., Weller W., Shuguang J., Guo K. (2016). Jovens universitários em um mundo em transformação: uma pesquisa sino-brasileira. Brasília/DF.

[5] Andriola W.B. (2011). Twelve reasons for the adoption of the national examination for secondary education (ENEM) by federal institutions of higher education (IFES). Ensaio: Avaliação e Políticas Públicas em Educação.

[6] Martins A.C.P. (2002). Ensino superior no Brasil: da descoberta aos dias atuais. Acta Cir Bras.

[7] Brazil (1961).

[8] Brazil (1988).

[9] Brazil (1996).

[10] Carvalho M.M.D.E., Waltenberg F.D. (2015). Desigualdade de oportunidade no acesso ao ensino superior no Brasil: uma comparação entre 2003 e 2013. Economia Aplicada.

[11] Diaz M.D.M. (2012). (Des)Igualdades de oportunidades no ensino médio brasileiro: Escolas públicas e privadas. Revista EconomiA.

[12] Instituto Nacional de Estudos e Pesquisas Educacionais Anísio Teixeira (INEP) (2025). Higher Education Census. https://www.gov.br/inep/pt-br/areas-de-atuacao/pesquisas-estatisticas-e-indicadores/censo-da-educacao-superior/resultados.

[13] Brazil (1998).

[14] Brazil (2009).

[15] Brazil (2001).

[16] Brazil (2022).

[17] Brazil (2005).

[18] Brazil (2022).

[19] Brazil. Decree No. 6,096 of April 24, 2007 (2007). Establishes the Programme for Restructuring and Expansion of Federal Universities (REUNI).

[20] Brazil (2010).

[21] Brazil (2012).

[22] Machado C., Szerman C. (2021). Centralized college admissions and student composition. Econ Educ Rev.

[23] Demenech L.M., Dumith S.C., Ferreira L.S. (2019). How far can you go? Association between illicit drug use and academic migration. J Bras Psiquiatr.

[24] Oliveira A.L.M. (2021). Profile of undergraduate students between 2001 and 2015: a review. Avaliação: Revista da Avaliação da Educação Superior (Campinas).

[25] Associação Nacional dos Dirigentes das Instituições Federais de Ensino Superior (Andifes) (2021).

[26] Instituto Brasileiro de Geografia e Estatística (IBGE) (2023).

[27] Oliveira A.L.M., Pochmann M., Rossi P. (2022). Interrupted inclusion? Higher education in Brazil in the beginning of the 21st century. Econ e Soc.

[28] Campbell F., Blank L., Cantrell A. (2022). Factors that influence mental health of university and college students in the UK: a systematic review. BMC Public Health.

[29] Evans-Lacko S., Thornicroft G. (2019). Viewpoint: WHO World Mental Health Surveys International College Student initiative: Implementation issues in low- and middle-income countries. Int J Methods Psychiatr Res.

[30] Rossi P., Oliveira A.L.M., Arantes F., Dweck E. (2019). Fiscal austerity and the financing of education in Brazil. Educ Sociedade.

[31] Araújo M.A.D., Macedo M.N. (2022).

[32] Knobel M., Leal F. (2021). The tragedies of Brazilian higher education. Int High Educ.

[33] Statista (2025).

[34] Resende C., Dweck E. (2022).

[35] Borges B., Resende C., Pires M. (2023). Arcabouço Constitucional: modificações recentes e como isso condicioona a nov regra fiscal em preparação pelo Governo Federal. https://observatorio-politica-fiscal.ibre.fgv.br/politica-economica/outros/arcabouco-constitucional-modificacoes-recentes-e-como-isso-condiciona-nova.

[36] Bielschowsky C.E. (2020). Trends of precariousness in private higher education in Brazil. Revista Brasileira de Política e Administração da Educação.

[37] Mello S.L.M., Meiriño M.J., Leal Filho W., Sampaio T.N.D.R. (2023). Promoting inclusion and equity in higher education: is this the role of distance learning in Brazil?. Ens Avaliação Políticas Públicas em Educ.

[38] World Health Organization (2025).

[39] Healthy Minds Network (2026). Healthy minds study among colleges and universities, 2013-2025. https://healthymindsnetwork.org/research/data-for-researchers.

[40] Lipson S.K., Zhou S., Abelson S. (2022). Trends in college student mental health and help-seeking by race/ethnicity: findings from the national healthy minds study, 2013-2021. J Affect Disord.

[41] Demenech L.M., Oliveira A.T., Neiva-Silva L., Dumith S.C. (2021). Prevalence of anxiety, depression and suicidal behaviors among Brazilian undergraduate students: a systematic review and meta-analysis. J Affect Disord.

[42] Soares S.J.B., Fernandes C.F.G., Tabalipa R. (2022). Common mental disorders among medical students: systematic review and meta-analysis of Brazilian studies. Sao Paulo Med J.

[43] Nguyen A., Tran L., Duong B.-H. (2023). Higher education policy and management in the post-pandemic era. Policy Futur Educ.

[44] Zhang L., Li Q., Duffy P., Zhang Z., Xu J., Cai J. (2024). Assessing the impact of the COVID-19 pandemic on graduate learning experiences in higher education: insights and recommendations. SAGE Open.

[45] Camargo Júnior E.B., Noivo I.S., Gouvea T.C.C., Fernandes M.N.F., Gherardi-Donato E.C.S. (2024). Depression and substance use among Brazilian university students during the COVID-19 pandemic. J Psychoactive Drugs.

[46] Schuch H.S., Cademartori M.G., Dias V.D. (2023). Depression and anxiety among the University community during the Covid-19 pandemic: a study in Southern Brazil. An Acad Bras Cienc.

[47] Demenech L.M., Neiva-Silva L., Brignol S.M.S. (2023). Suicide risk among undergraduate students in Brazil in the periods before and during the COVID-19 pandemic: results of the SABES-Grad national survey. Psychol Med.

[48] da Silva Bandeira B.E., dos Santos Júnior A., Dalgalarrondo P., de Azevedo R.C.S., Celeri E. (2022). Nonsuicidal self-injury in undergraduate students: a cross-sectional study and association with suicidal behavior. Psychiatry Res.

[49] Demenech L.M., Paulitsch R.G., Silva S.L., Martins A.C.R., Neiva-Silva L., Dumith S.C. (2023). Social determinants of quality of life among undergraduate students and their association with suicide risk. Sci Med.

[50] Fonseca L.B., Pereira L.P., Rodrigues P.R.M. (2022). Incidence of depressive symptoms and its association with sociodemographic factors and lifestyle-related behaviors among Brazilian university students. Psychol Health Med.

[51] da Silva Júnior A.E., de Lima Macena M., de Oliveira A.D.S., Praxedes D.R.S., de Oliveira Maranhão Pureza I.R., Bueno N.B. (2022). Racial differences in generalized anxiety disorder during the COVID-19 pandemic among Brazilian university students: a national survey. J Racial Ethn Health Disparities.

[52] Demenech L.M., Neiva-Silva L., Brignol S.M.S. (2023). A Study on the Health and Wellness of Undergraduate Students (SABES-Grad): methodological aspects of a nationwide multicenter and multilevel study overlapped with the Covid-19 pandemic. Trends Psychiatry Psychother.

[53] Lima J.D., Plácido J., Andrade B. (2025). Intersectionality and mental health in university students: a jeopardy index approach. Rev Saude Publica.

[54] Collins P.H. (2015). Intersectionality's definitional dilemmas. Annu Rev Sociol.

[55] Crenshaw K. (1991). Mapping the margins: intersectionality, identity politics, and violence against women of color. Stanford Law Rev.

[56] Demenech L.M., Neiva-Silva L., Antochevis A.F., Almeida T.R., Dumith S.C. (2023). Perceived stress among undergraduate students: associated factors, influence of the ENEM/SiSU model, and its possible consequences on health. J Bras Psiquiatr.

[57] Flesch B.D., Houvèssou G.M., Munhoz T.N., Fassa A.G. (2020). Major depressive episode among university students in Southern Brazil. Rev Saude Publica.

[58] Andleeb H., Moltrecht B., Gayer-Anderson C. (2024). Age-at-migration, ethnicity and psychosis risk: findings from the EU-GEI case-control study. PLOS Ment Health.

[59] McManus S., Gunnell D. (2020). Trends in mental health, non-suicidal self-harm and suicide attempts in 16-24-year old students and non-students in England, 2000-2014. Soc Psychiatry Psychiatr Epidemiol.

[60] Grelle K., Shrestha N., Ximenes M. (2023). The generation gap revisited: generational differences in mental health, maladaptive coping behaviors, and pandemic-related concerns during the initial COVID-19 pandemic. J Adult Dev.

[61] Ebert D.D., Buntrock C., Mortier P. (2019). Prediction of major depressive disorder onset in college students. Depress Anxiety.

[62] Comptom M.T., Shim R.S. (2015).

[63] Associação Nacional dos Dirigentes das Instituições Federais de Ensino Superior (Andifes) (2022). Andifes debate saúde nas Universidades Federais. https://www.andifes.org.br/?p=94640.

[64] Conselho Nacional de Desenvolvimento Científico e Tecnológico (CNPq) (2023). CNPq/MCTI 2023 public call – support for the creation of a Brazilian Research, Development, and Innovation network in mental health. http://memoria2.cnpq.br/web/guest/chamadas-publicas?p_p_id=resultadosportlet_WAR_resultadoscnpqportlet_INSTANCE_0ZaM&idDivulgacao=11588&filtro=abertas&detalha=chamadaDetalhada&id=47-2320-8849.

[65] Pacheco J.P., Giacomin H.T., Tam W.W. (2017). Mental health problems among medical students in Brazil: a systematic review and meta-analysis. Braz J Psychiatry.

[66] World Health Organization (2025).

[67] Osborn T.G., Li S., Saunders R., Fonagy P. (2022). University students' use of mental health services: a systematic review and meta-analysis. Int J Ment Health Syst.

[68] Gaiotto E.M.G., Trapé C.A., Campos C.M.S. (2021). Response to college students' mental health needs: a rapid review. Rev Saude Publica.

[69] Duffy A. (2023). University student mental health: an important window of opportunity for prevention and early intervention. Can J Psychiatry.

[70] Worsley J.D., Pennington A., Corcoran R. (2022). Supporting mental health and wellbeing of university and college students: a systematic review of review-level evidence of interventions. PLoS One.

[71] Singla D.R., Kohrt B.A., Murray L.K., Anand A., Chorpita B.F., Patel V. (2017). Psychological treatments for the world: lessons from Low- and middle-income countries. Annu Rev Clin Psychol.

[72] Patel V. (2022). Scale up task-sharing of psychological therapies. Lancet.

[73] Brown A.D., Ross N., Sangraula M., Laing A., Kohrt B.A. (2023). Transforming mental healthcare in higher education through scalable mental health interventions. Glob Ment Health (Camb).

[74] Bloc L., de Araújo J.L., Leite J.M. (2022). Virtual clinical listening groups for psychological intervention with university students in the COVID-19 pandemic. Front Psychiatry.

[75] Prado A.D.S., Kohls E., Baldofski S., Rummel-Kluge C., Freitas J.D.L. (2023). Acceptability and feasibility of online support groups for mental health promotion in Brazilian graduate students during the COVID-19 pandemic: longitudinal observational study. JMIR Form Res.

